# The utility of Glaucoma Information Cards for grasping glaucoma subtypes: a single-center pilot study

**DOI:** 10.1186/s40780-025-00414-9

**Published:** 2025-02-04

**Authors:** Yukinori Yamada, Tetsuya Murase, Emiko Mashida, Yuki Kakeda, Yuuka Shibata, Hiroaki Matsuo

**Affiliations:** Department of Pharmaceutical Services, Hiroshima University Hospital, 1-2-3 Kasumi, Minami-Ku, Hiroshima, 734-8551 Japan

**Keywords:** Open-angle glaucoma, Angle-closure glaucoma, Anticholinergic drug, Proper use

## Abstract

**Background:**

In Japan, anticholinergic drugs were contraindicated for glaucoma, regardless of subtype. The contraindication in patients with open-angle glaucoma (OAG) led to excessive medication restrictions because anticholinergic drugs are associated with a low risk of acute glaucoma attacks in OAG. To eliminate this problem, the contraindications of anticholinergic drugs were reduced for types from all glaucoma to angle closure glaucoma (ACG) in 2019. This change requires differentiation of OAG from ACG for the proper use of drugs. The Japan Ophthalmologists Association created Glaucoma Information Cards to share glaucoma subtypes and restricted the medication list. However, only a few studies have investigated the efficacy of Glaucoma Information Cards. In this study, we evaluated the benefits of identifying glaucoma subtypes prior to admission by distributing Glaucoma Information Cards to patients through pharmacists.

**Methods:**

Pharmacists distributed Glaucoma Information Cards to patients whose glaucoma subtype was unclear at the time of the preadmission pharmacist interview and asked them to have the treating ophthalmologist fill out the glaucoma subtype data. We collected patient data, hospitalized clinical department, status of realization of glaucoma subtypes, methods of realization of glaucoma subtypes, and usage of anticholinergic drugs from medical records.

**Results:**

First, we investigated how hospital pharmacists identify the type of glaucoma. Pharmacists were able to determine the glaucoma type of most patients through medical records, patient interviews, and the Glaucoma Information Card. We analyzed the records of patients who were scheduled for ophthalmology visits prior to admission and had been given Glaucoma Information Cards. Eleven of the 13 patients brought the Glaucoma Information Card to the hospital, which was analyzed by an ophthalmologist. Two patients were administered anticholinergic drugs without restriction because of proven OAG according to the Glaucoma Information Card. One patient was administered cibenzoline for atrial fibrillation. Another patient received isosorbide dinitrate for coronary angiography. These cases indicate that the distribution of Glaucoma Information Cards can help avoid unnecessary medication restrictions in patients with OAG.

**Conclusions:**

Our results suggest that the distribution of the Glaucoma Information Cards is useful for proper drug use in patients with glaucoma.

## Background

Glaucoma involves characteristic changes in the neuroretinal rim tissue of the optic nerve head and progressive loss of retinal ganglion cells accompanied by visual field constriction [1]. There are several types of glaucoma, such as open-angle glaucoma (OAG) and angle closure glaucoma (ACG). Patients with ACG are at high risk of acute glaucoma attack due to anticholinergic drug administration. Thus, administration of anticholinergic drugs is restricted in these patients. However, patients with OAG have a low risk of acute glaucoma attacks. In Europe and the United States, anticholinergic drugs are considered safe for patients with OAG, and there are no contraindications for their use in glaucoma [2]. Even so, anticholinergic drugs had been contraindicated for glaucoma regardless of the subtype in Japan. This contraindication has led to unnecessary medication restrictions for OAG patients, despite the benefits of safely administration of anticholinergic drugs [3]. To resolve this excessive drug restriction, the Japanese Ophthalmological Society requested a change in the contraindications of anticholinergic drugs in the package insert, resulting in the revision of the contraindication for types ranging from glaucoma to ACG in 2019 (Pharmaceuticals and Medical Devices Safety Information No. 364, p10-13,2019. https://www.pmda.go.jp/files/000230335.pdf). This change allows the administration of anticholinergic drugs to patients with OAG and reduces excessive drug restriction.

**Fig. 1:**
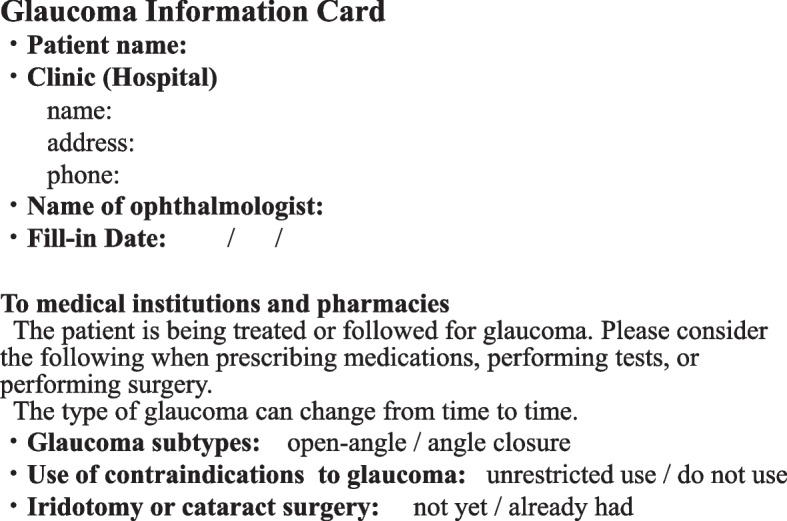
Glaucoma Information Cards

Recently, drug-induced acute glaucoma attacks have been reported both in Japan and other countries, and the requirement for the accurate identification of glaucoma subtypes has increased [4]. Older glaucoma patients at times have multiple diseases, consult different non-ophthalmologic clinics, and are prescribed a variety of drugs. These patients are often hospitalized for various purposes, including chemotherapy, surgery, and checkups, and are likely to be administered various drugs, such as anticholinergic drugs. The administration of various drugs poses a serious risk of acute glaucoma attacks, and accurately identifying glaucoma subtypes is valuable strategy to mitigate this risk [5]. However, only a few studies have addressed glaucoma categorization in clinical settings, which requires further clarification. To facilitate the accurate communication of glaucoma subtypes and medication restrictions among physicians, pharmacists, and patients, the Japan Ophthalmologists Association developed a Glaucoma Information Card (Fig. 1). This card is distributed by primary care ophthalmology centers. The card includes records of glaucoma subtypes and related drug restrictions. In other hospitals and pharmacies, medical staff can obtain information about glaucoma through this card. This card has also been reported to improve patients’ awareness of the disease [6]. We expected that using the Glaucoma Information Card would contribute to the realization of accurate glaucoma subtypes and drug restriction. We distributed Glaucoma Information Cards to patients through pharmacists at Hiroshima University Hospital (HUH), and requested the consultation ophthalmologist to record the types of glaucoma during prehospital interviews on July 1, 2022.

In this study, we investigated how pharmacists in hospitals identify glaucoma subtypes before and after distribution of the Glaucoma Information Card by pharmacists in HUH. We also analyzed the efficacy of Glaucoma Information Card distribution for accurate communication of glaucoma subtypes and related drug limitations by investigation of the effect on understanding of glaucoma subtype and appropriateness of the pharmacotherapy such as unnecessary restriction or not after hospital admission.

## Methods

### Study population

This study included patients admitted to Hiroshima University Hospital (HUH) who were prescribed glaucoma medications. The study consisted of two phases:

1. From March 15 to April 15, 2021, focusing on methods for identifying glaucoma types and the usage status of anticholinergic drugs.
2. From July 1 to December 31, 2022, focusing on identifying glaucoma types and anticholinergic drug usage by pharmacists during pre-admission interviews and the distribution of Glaucoma Information Cards.

**Fig. 2:**
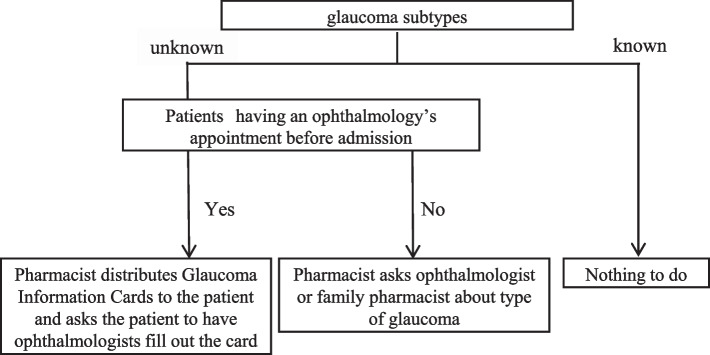
The distribution flow of Glaucoma Information Cards in pre-admission pharmacist interviews

The distribution process of the Glaucoma Information Card is outlined in Fig. 2. Patients who used eye drops for glaucoma during the pharmacists’ prehospital interviews at HUH were candidates for Glaucoma Information Card distribution. The cards were distributed to those with unknown glaucoma subtypes at prehospital interviews and scheduled ophthalmology consultation before hospital admission (Fig. 2).

### Data collection and evaluation of usefulness of the Glaucoma Information Card

We collected patient data from the medical records, hospitalized clinical department, identification status of glaucoma subtypes, methods of realization of glaucoma subtypes (medical records, patient’s recognition, Glaucoma Information Card, drug notebook, family pharmacist inquiry, or ophthalmologist consultation), and the use of anticholinergic drugs contraindicated for ACG but not for OAG. The usefulness of using Glaucoma Information Cards was evaluated based on the understanding of the glaucoma type and appropriateness of the pharmacotherapy such as unnecessary restriction or not.

## Results

### Methods for identifying glaucoma types and the usage status of anticholinergic drugs by pharmacists before the issuance of Glaucoma Information Cards

**Fig. 3:**
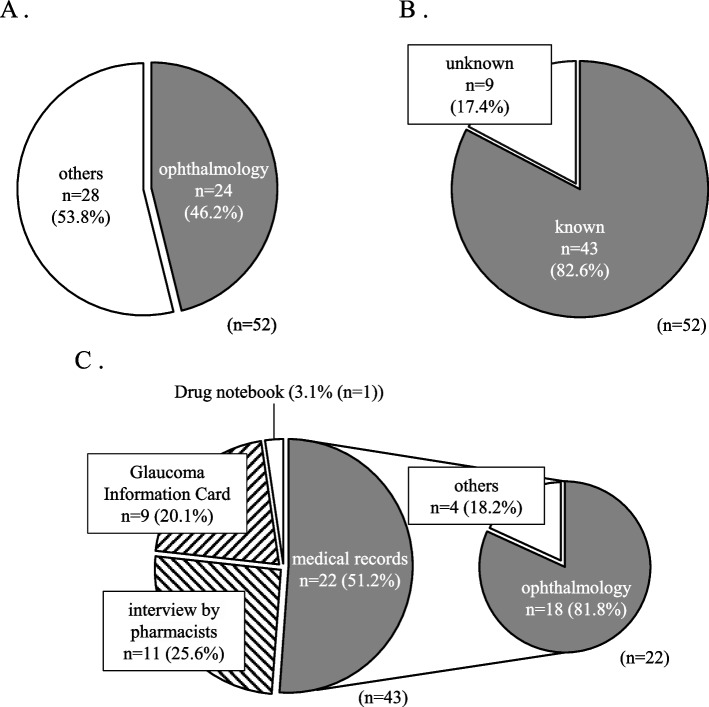
Status and methods of identifying the glaucoma subtypes at hospital admission. **A** Hospitalized department (*n* = 52). **B** Status of glaucoma subtypes identification (*n* = 52). **C** Methods of identifying glaucoma subtypes identification (*n* = 43) and the hospitalized department of patient whose glaucoma subtypes identified from medical records (*n* = 22)

Between March 15, and April 14, 2021, 52 patients who underwent glaucoma treatment were admitted to our hospital. Of these, 24 patients (46.2%) were admitted for ophthalmological treatment and the remaining 28 patients (53.8%) were admitted to other departments, such as gastroenterology, metabolism, or emergency and intensive care units (Fig. 3A). As shown in Fig. 3B, the pharmacists determined the type of glaucoma in 43 patients in the admission interviews. Of these, 41 patients had OAG and 2 patients had ACG. The methods by which the type of glaucoma was identified in these 43 patients included information from the medical records for 22 patients (51.2%), patient interviews for 11 patients (25.6%), Glaucoma Information Card for nine patients (20.1%), and drug notebook for one patient (3.1%) (Fig. 3C). Among the 22 patients whose glaucoma type was identified through medical records, 18 were admitted for ophthalmological treatment, and the remaining four were admitted to other departments. Of the 11 patients whose glaucoma type was identified through patient interviews, only two correctly recognized their condition. The remaining nine patients reported not being informed by their ophthalmologists about any medication restrictions related to glaucoma. For the nine patients whose glaucoma type was not identified during admission interviews, pharmacists assessed the necessity of determining their glaucoma type based on planned treatments and care. For patients deemed necessary, the responsible pharmacist confirmed the glaucoma type and medication restrictions. This verification was performed in 3 patients after admission through phone consultations with their ophthalmologists. In contrast, five patients confirmed that determine of glaucoma types was not necessary. Among these patients, four underwent glaucoma surgery scheduled on the day of admission, and post-surgery, there were no restrictions on medications such as anticholinergic drugs. One patient, admitted with a decreased level of consciousness and later issued a Do Not Attempt Resuscitation (DNAR) order, was deemed unnecessary for drug restriction. Additionally, one patient admitted due to anaphylactic shock from contrast media was urgently treated with d-chlorpheniramine before the glaucoma type was confirmed. This patient did not exhibit glaucoma attacks following administration and was discharged the day after improvement.

### Identification of glaucoma type and anticholinergic drug use by pharmacists during pre-admission interviews

**Fig. 4:**
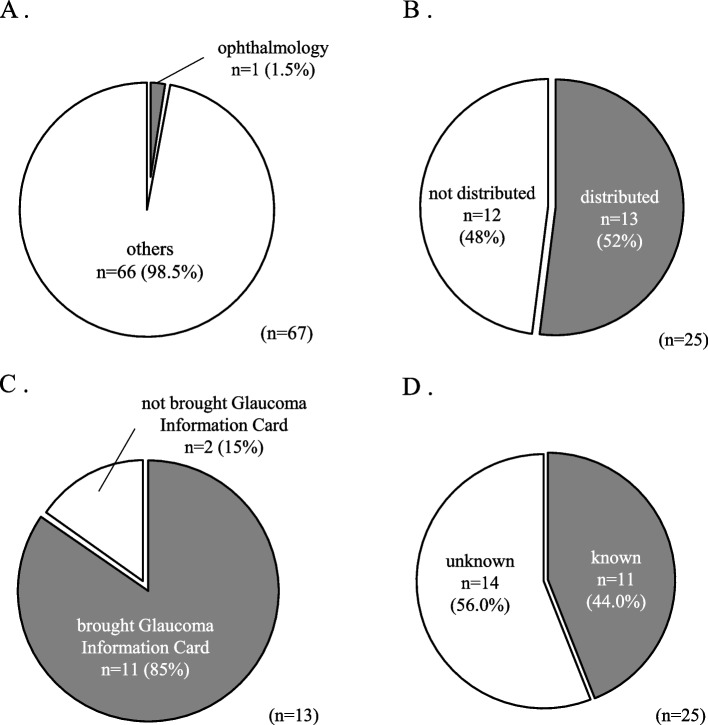
Distribution status of Glaucoma Information Cards at pre-admission pharmacist interviews and carrying and the status of understanding of glaucoma subtypes at the time of admission. **A** Hospitalized department (*n* = 67). **B** Distribution status of Glaucoma Information Cards at pre-admission pharmacist interviews (*n* = 25). **C** Carrying status of Glaucoma Information Card at the time of hospital admission (*n* = 13). **D** Status of understanding of glaucoma subtypes at pre-admission interview by pharmacist (*n* = 25)

Over the six-month period starting July 1, 2022, pre-admission pharmacist interviews were conducted with 67 patients. One patient (1.5%) consulted the ophthalmology and the remaining 66 patients (98.5) consulted other clinical departments (Fig. 4A). Among these, the glaucoma type was identified in 42 patients (62.7%), while 25 patients (37.3%) had an unknown glaucoma type or unclear medication restrictions. Among these 25 patients, 13 patients (52%) visited their ophthalmologists before admission and were distributed Glaucoma Information Cards with a request for their ophthalmologists to fill in the necessary information (Fig. 4B). Glaucoma Information Cards were not distributed to 12 patients (48%) because they did not have an opportunity to visit their ophthalmologists before admission. At the time of admission, 85% (11 of 13) of these patients brought their completed Glaucoma Information Cards, enabling accurate identification of their glaucoma types (Fig. 4C). The distribution of the Glaucoma Information Card facilitated the identification of glaucoma types, with 44.0% (11 of 25) of patients whose glaucoma types unknown before distribution successfully identified (Fig. 4D). This result suggested that Glaucoma Information Cards are useful for identifying glaucoma types. There were notable cases in which Glaucoma Information Cards significantly impacted patient care (Table 1). For example, one patient with OAG, identified by Glaucoma Information Cards, developed atrial fibrillation after an abdominal aortic replacement and was administered cibenzoline, an anticholinergic drug. Another patient, whose glaucoma disease subtype was identified by Glaucoma Information Cards, was administered isosorbide dinitrate, which is contraindicated for ACG, during coronary angiography. These patients were administered anticholinergic drugs appropriately because glaucoma subtypes were confirmed using Glaucoma Information Cards. Without the Glaucoma Information Card, the glaucoma type would have remained unknown, and these patients might have been deprived of the necessary medications owing to the potential risk of ACG. The issuance of the Glaucoma Information Cards confirmed that these patients did not have ACG, thus enabling the appropriate administration of anticholinergic drugs and other necessary medications. Additionally, five patients whose glaucoma disease subtypes were identified by Glaucoma Information Cards continued receiving anticholinergic drugs they had brought from home or those prescribed for insomnia during their hospital stay. Of the 13 patients who received Glaucoma Information Cards, two were identified as having ACG and two were identified as having OAG. However, there was no change in their medication as there were no planned medications contraindicated for ACG. Among the 12 patients who could not be issued Glaucoma Information Cards, the glaucoma type was confirmed at the time of admission in 10 patients (Table 2). This included one patient who was contacted by an ophthalmologist and another who received a consultation from our hospital's ophthalmologist after admission. For the remaining 2 patients whose glaucoma type could not be determined, no anticholinergic drugs were planned during their hospital stay.

**Table 1:** Usage of anticholinergic drugs for OAG patients whose glaucoma type was identified by the Glaucoma Information Card

**Usage of anticholinergic drugs or not**		**patients**
**Yes**	cibenzoline for atrial fibrillation	**1**
	isosorbide dinitrate for coronary angiography	**1**
	continued zolpidem brought in by the patient	**1**
	continued of eszopiclone instructions for insomnia	**4**
**No**		**2**
	**total**	**9**

**Table 2:** Identification of glaucoma disease types and intervention for patients without Glaucoma Information Cards

Glaucoma disease type conformation	intervention	patients
**Yes**	consultation with their ophthalmology	**1**
	consultation with our hospital's ophthalmology after admission	**1**
	confirmation from medical charts	**2**
	conformation from patient’s interviews	**5**
	inference from drug brought in	**1**
**No**	no plan to use anticholinergic drugs during hospitalization	**2**
	**total**	**12**

## Discussion

Identifying glaucoma types and medication restrictions is crucial for providing appropriate pharmacotherapy to glaucoma patients. According to a report by Masuda et al., pre-admission interviews with patients scheduled for endoscopic procedures or surgeries revealed that 17.4% of those undergoing endoscopic procedures and 15.2% of those undergoing surgeries had received medication restriction instructions from their ophthalmologists [7]. These restrictions were confirmed by pharmacists through written communications with ophthalmologists at other facilities, as obtaining accurate information directly from patients was deemed very difficult. However, in daily clinical practice, pharmacists often rely on patient interviews and confirmatory methods. Therefore, it is desirable for patients to accurately understand their glaucoma types and medication restrictions.

During the admission process, glaucoma types was identified in approximately 80% of patients undergoing glaucoma treatment, regardless of their own recognition of the condition. The most common method of identification was through the hospital's medical records, which accounted for 51.2% of the cases. All patients were either admitted for ophthalmic treatment or were regular patients in the ophthalmology department of the hospital. Conversely, patients not regularly seen by our hospital's ophthalmology department lacked records of their glaucoma types in medical records or referral letters, which often only mentioned a history of glaucoma without specifying the type. Therefore, it is difficult to identify glaucoma types from medical records, unless the patient is regularly observed by our hospital's ophthalmology department. The second most common method of identification was patient interviews, which accounted for 25.6% of cases. However, this study found that many patients were not informed of medication restrictions related to glaucoma by their ophthalmologists, and only two patients could accurately identify their glaucoma type, raising concerns about the reliability of patient-reported information The third most common method was the Glaucoma Information Card, which accounted for 20.1% of the cases. The Glaucoma Information Card is a tool that allows the sharing of information about glaucoma by having ophthalmologists record and distribute the card, which patients can then present to healthcare providers. This makes it easy to identify the type of glaucoma, regardless of the department or medical institution to which the patient was admitted. For patients whose glaucoma type could not be identified during the pharmacist admission interview, individual pharmacists determined the necessity of identifying the glaucoma type based on the patient's treatment plan and medical needs. In some cases, the pharmacist contacted the patient's ophthalmologist by phone, although this method could have been delayed if admission occurred outside the ophthalmologist’s office hours. The glaucoma type could not be identified in some patients because of a decreased level of consciousness at the time of admission or the urgent nature of their condition, which necessitated the use of anticholinergic drugs before confirming the glaucoma type. These patients were judged not to require identification of their glaucoma type based on their treatment plans. However, if these patients carried Glaucoma Information Cards, it would have been possible to select the appropriate medications based on their glaucoma type. Therefore, Glaucoma Information Cards, unaffected by the patient’s awareness or consciousness level, or the clinical environment, are highly useful for pharmacists in identifying glaucoma types and medication restrictions at the time of admission.

In this study, more than 80% of patients who received Glaucoma Information Cards during pre-admission pharmacist interviews had their glaucoma type recorded by their ophthalmologists, allowing pharmacists to identify their glaucoma type at the time of admission. This improved the accuracy of glaucoma subtype identification during hospitalization. In patients whose glaucoma type was identified through Glaucoma Information Cards, examples of cases where medication restrictions were not necessary included the administration of cibenzoline succinate to a patient who developed atrial fibrillation after abdominal aorta replacement, and the administration of isosorbide dinitrate, which is contraindicated for ACG, to a patient undergoing coronary angiography (Table 1). These cases demonstrate that identifying glaucoma types using Glaucoma Information Cards influenced pharmacotherapeutic decisions. Additionally, the continuation of zolpidem tartrate tablets, which the patient brought from home, and the prescription of eszopiclone for insomnia were made possible by confirming that the patient had OAG, thus avoiding unnecessary restrictions on anticholinergic drugs. These cases also suggest that the Glaucoma Information Card prevents unnecessary changes in pharmacotherapy. These results suggest that distributing Glaucoma Information Cards during preadmission pharmacist interviews is effective in identifying the type of glaucoma at the time of admission and helps avoid unnecessary medication restrictions for patients with OAG.

In this study, Glaucoma Information Cards were not distributed to patients without scheduled ophthalmologist’s visits before admission. This decision was made by the pharmacist to avoid placing additional burdens on patients who did not have scheduled ophthalmologist visits and to ensure accurate glaucoma types. For these patients, the latest glaucoma type was verified by a chart review after admission, and medication strategies were considered. However, for two patients whose glaucoma type was unknown and who were likely to require anticholinergic medication after admission, the pharmacists contacted the ophthalmologists. Additionally, some patients had glaucoma as confirmed by the hospital’s ophthalmology department after admission. For patients who do not have scheduled ophthalmologist visits before admission or have not undergone pre-admission interviews, it is necessary to identify the glaucoma type after admission. To identify glaucoma types in these patients, appropriate use of the Glaucoma Information Card and collaboration of medical staff are essential. As Glaucoma Information Cards are filled out and issued by ophthalmologists, pharmacists do not record this information. Therefore, it is essential to develop methods for sharing identified glaucoma types and post-surgery medication restrictions with other healthcare providers and outpatient pharmacies. Sharing electronic medical record information is anticipated as part of the ongoing digital transformation in the medical field.

## Conclusion

This study focused on the use of Glaucoma Information Cards and showed that the intervention of hospital pharmacists in distributing the cards during the pre-admission pharmacist’s interview was effective in identifying the type of glaucoma at the time of admission and avoiding unnecessary medication restrictions for patients with OAG.

## Data Availability

The datasets used and/or analyzed during the current study are available from the corresponding author upon reasonable request.

## Abbreviations

OAG: Open-Angle Glaucoma
ACG: Angle Closure Glaucoma
HUH: Hiroshima University Hospital
DNAR: Do Not Attempt Resuscitation
